# Should I Stay or Should I Go? The Role of Motivational Climate and Work–Home Spillover for Turnover Intentions

**DOI:** 10.3389/fpsyg.2020.01107

**Published:** 2020-05-28

**Authors:** Karoline Hofslett Kopperud, Christina G. L. Nerstad, Anders Dysvik

**Affiliations:** ^1^Oslo Business School, Oslo Metropolitan University, Oslo, Norway; ^2^BI Norwegian Business School, Oslo, Norway

**Keywords:** turnover intention, perceived motivational climate, positive work–home spillover, negative work–home spillover, life-supportive workplaces

## Abstract

Emerging trends in the workforce point to the necessity of facilitating work lives that foster constructive and balanced relationships between professional and private spheres in order to retain employees. Drawing on the conservation of resources theory, we propose that motivational climate influences turnover intention through the facilitation of work–home spillover. Specifically, we argue that employees working in a perceived mastery climate are less likely to consider voluntarily leaving their employer because of increased positive–and reduced negative—work–home spillover experiences. We further argue that employees working in a perceived performance climate are more likely to consider voluntarily leaving their employer because of reduced positive—and increased negative—work–home spillover experiences. In a cross—lagged survey of 1074 employees in a Norwegian financial-sector organization, we found that work–home spillover partly mediates the relationship between a perceived motivational climate and turnover intention. Specifically, mastery climates seem to facilitate positive—and reduce negative—spillover between the professional and private spheres, which in turn decreases employees’ turnover intention. Contrary to our expectations, a perceived performance climate slightly increased both positive and negative work-home spillover, however increasing employees’ turnover intention. We discuss implications for practice and future research.

## Introduction

The number of both dual-earner couples and single parents in the workforce has increased. At the same time, younger workers report augmented work pressures and place greater value on non-work activities (Twenge et al., 2010). Due to a desire to focus on both work and non-work activities, it is important that employees perceive a positive interaction between work and non-work, thereby avoiding a feeling of conflict between life arenas (Mauno et al., 2015). Individuals who feel that their work life generates positive resources that benefit areas of their non-work life may experience increased well-being and motivational outcomes (Mauno et al., 2015). In turn, such positive consequences could influence their intentions to stay with their current employer. On the other hand, individuals who feel that their work life generates negative experiences, such as stress or fatigue, may want to withdraw from their work role to focus more of their resources on non-work activities. Intention to withdraw is costly for organizations as turnover intention has been found to be one of the most important predictors of actual turnover (Griffeth et al., 2000). Turnover intention has been linked to increased costs (Collins and Smith, 2006); reduced stability, quality, and consistency of the services that organizations provide (Trevor and Nyberg, 2008); and reduced customer satisfaction (Lin and Chang, 2005). Therefore, facilitating workplaces that foster positive spillover and reduce negative spillover between employees’ professional and private spheres in order to retain them is among the salient challenges facing organizations today (Lobel et al., 1999; Giddens, 2000; Greenhaus and Powell, 2006; McNall et al., 2010). Work–home spillover has proven vital to reduce turnover intention (e.g., Russo and Buonocore, 2012; Mihelic, 2014; Mauno et al., 2015). Considerable research has focused on how to facilitate life supportive work places in terms of both organizational practices and family supportive work climates (e.g., Lapierre et al., 2008; Aryee et al., 2013; Rofcanin et al., 2016). However, less attention has been paid to how other work climates—beyond that of family support—influence spillover between work and non-work activities. Thus, the purpose of this study was to extend existing work that has been largely limited to the role of family supportive climates in fostering positive and reducing negative spillover between work and non-work in order to reduce turnover intentions. This is an interesting avenue of research because work–home spillover should extend beyond family life in order to have impact on the broader workforce. Employees, regardless of their marital and parental status, appear equally motivated to blend work and non-work activities in satisfying ways (Twenge et al., 2010). Therefore, work climates other than those pertaining to a family focus may generally motivate or demotivate employees to stay with their employer due to their life-supporting elements, or lack thereof (Greenhaus and Powell, 2017). Specifically, our aim in this study was to investigate the mediating role of work–home spillover between a perceived motivational work climate and turnover intentions among all of today’s employees.

Achievement goal theory (AGT) distinguishes between two goal-reward structures, a mastery motivational climate and a performance motivational climate (Ames, 1992b). In a mastery motivational climate, learning, growth, effort, and cooperation define success and are therefore valued (Nicholls, 1989; Ames, 1992b). In contrast, a performance climate is seen as controlling and primarily focuses on relative ability, social comparisons, and rivalry among employees, as well as valuing demonstrations of superior ability by employees (Ames, 1992a; Roberts and Nerstad, 2020).

A perceived mastery climate has previously been found to predict adaptive individual outcomes, including intentions to stay, work engagement, autonomy, felt trust, knowledge sharing, innovative work behavior, and work performance. A perceived performance climate, on the other hand, has been found to predict maladaptive outcomes such as turnover intentions, controlled motivation, knowledge hiding, reduced performance, and felt trust (Ntoumanis and Biddle, 1999b; Valentini and Rudisill, 2006; Nerstad et al., 2013, 2018; Černe et al., 2017).

One important aspect of a mastery climate is that leaders and employees share responsibility for making choices, giving directions, and monitoring work (cf. Valentini and Rudisill, 2006). Thus, because employees are allowed to establish priorities, pace their learning process, develop self-leadership, and employ self-regulatory strategies at work (cf. Ames, 1992a; Valentini and Rudisill, 2006), they acquire more resources, which should increase positive and reduce negative work–home spillover. We propose that mastery climate conditions facilitate the provision of sufficient and valuable resources that will motivate individuals to stay with their current employer. We expect this relationship to be mediated by increased positive and reduced negative work–home spillover because a mastery climate should represent important elements in what Greenhaus and Powell (2017) referred to as life-supportive work climates. On the other hand, in a performance climate, an employee’s opportunity to receive rewards or achieve goals is reduced when other colleagues are successful (Ames and Ames, 1984). Thus, employees are constantly involved in rivalry and forced social comparisons, and their progress is judged based on normative standards. We propose that this is likely to undermine employees’ resources—particularly at times when they are not at the top of their game—which could reduce positive and increase negative work–home spillover and thereby increase their intention to quit.

Work–home spillover is defined as the effects of work and home on one another “that generate similarities between the two domains” (Edwards and Rothbard, 2000, p. 180) and has been shown to be an important variable to reduce turnover intentions (e.g., Russo and Buonocore, 2012; Mihelic, 2014; Mauno et al., 2015). Although some studies have found contradictory results concerning the relationship between positive spillover and turnover intentions (e.g., Gordon et al., 2007; Moen et al., 2011), these studies have relied on either older or younger samples (Gordon et al., 2007; Moen et al., 2011). Furthermore, positive and negative spillover each seem to have different effects on outcomes (Wayne et al., 2015). Whereas negative spillover typically relates to negative outcomes, positive spillover typically relates to positive outcomes (Aboobaker and Edward, 2019). By focusing on the outcomes of negative and positive spillover separately, one neglects that experiences of negative and positive spillover together constitute the experience of work–home spillover (Mauno and Rantanen, 2013; Rantanen et al., 2013; Aboobaker and Edward, 2019). Thus, a specific focus on both positive and negative spillover to evaluate their contrasting relative contribution to turnover intentions should facilitate a more accurate assessment of the pathway between work–home spillover and turnover intention (cf. Grzywacz and Marks, 2000).

Our theoretical point of view and empirical findings are timely and important for both the work–home literature and the turnover literature for several reasons. First, we extend the work–home literature beyond a focus on family supportive climates to capture work climates that have a positive impact (i.e., lower turnover intentions) or negative impact (i.e., increased turnover) on the broader workforce. Second, increased turnover intention has been found to be a consequence of negative work–home spillover (cf., Boyar et al., 2003; Spector et al., 2007; Carr et al., 2008; O’Neill et al., 2009). However, studies exploring the link between positive work–home spillover and turnover intention have provided somewhat mixed results, indicating a need to further investigate this issue (McNall et al., 2010). We do so by extending existing research on work–home spillover and turnover intention by including and contrasting the influence of both positive and negative spillover on turnover intention. Third, by exploring whether a perceived motivational climate influences work–home spillover, we aim to respond to the calls to enrich our knowledge about contextual influences on work–home spillover and its outcomes (Burke, 2012; Major and Germano, 2012; Ten Brummelhuis and Bakker, 2012). We argue that a mastery climate is particularly beneficial to the perceptions of work–home spillover because it may equip employees with a pool of resources that are relevant for addressing and potentially foster positive, while reducing negative, work–home spillover. We also argue that a perceived performance climate is detrimental to the perceptions of work–home spillover because it drains employees of resources, which will negatively spill over into their non-work arenas.

For practical purposes, we seek to offer insight into how managers can promote positive work environments that help employees to create and sustain healthy relationships among various life roles and reduce turnover in organizations. Our study may help practitioners to better understand the factors that drive turnover intention across the workforce. That is, practitioners could benefit from arranging for a work climate that is characterized by a sense of control/autonomy, cooperation, learning, and growth (Ames, 1992b). In turn, such a climate may enhance opportunities for positive work–home spillover and thereby reduce turnover intention. The insight gained from this study may help organizations improve individual and organizational functioning.

## Theory and Hypotheses

### The Perceived Motivational Climate and Turnover Intention

Employees process information from their work environments in terms of salient values and goals (Ames and Ames, 1984). In turn, these goals and values affect employees’ perceptions, self-evaluations, attributions, and beliefs about the preferred actions and strategies in their workplaces (cf. Ames and Ames, 1984). As a result, qualitatively different perceptions (i.e., mastery or performance oriented criteria of success) of the goal–reward structure, which is conceptualized as the motivational climate, emerge (Nerstad et al., 2013). According to traditional AGT (Ames, 1992a; Nicholls, 1989), the motivational climate is defined as the extant criteria of success and failure that are emphasized in the workplace’s procedures, policies, and practices (Nerstad et al., 2013). Because the motivational climate places emphasis on individual interpretation, meaning, and experience, it represents a psychological climate (Ames, 1992a; Parker et al., 2003).

According to AGT, an important aspect of the motivational climate is a mastery climate (Ames, 1992b). In a mastery climate, the emphasis is on employees’ effort rather than their ability; learning, mastery, skill development, and cooperation are valued (Ames, 1992a; Nerstad et al., 2013). When working in a mastery climate, employees are rewarded for individual improvement, progress, and mastery. They are recognized for their effort and hard work, provided with opportunities to improve and encouraged to view mistakes as an important part of learning, actively participate in decision-making, use self-management and -monitoring skills, and actively approach challenges (Ames, 1992a). The growth process leading to performance is more important than the end result being the sole emphasis (Černe et al., 2014). Existing empirical evidence has shown that this type of climate fosters adaptive outcomes that include greater autonomous motivation (Ntoumanis and Biddle, 1999b; Nerstad et al., 2018). Work environments that increase employees’ sense of intrinsic motivation should help reduce their turnover intention because employees who are concerned with learning and development, but experience a low sense of intrinsic motivation, tend to have higher turnover intention (Dysvik and Kuvaas, 2010). Indeed, the important link between intrinsic motivation and reduced turnover has been documented in a recent meta-analysis (Rubenstein et al., 2018). Organizational climates have also been shown to be an important predictor of turnover intentions, although there are few studies so far (Rubenstein et al., 2018). To the best of our knowledge, only one study has empirically tested the direct relationship between a mastery climate and turnover intention in a work setting (Nerstad et al., 2013). This study showed that a mastery climate reduced turnover because of the beneficial effects it had on work-life quality. Therefore, we hypothesize the following:

Hypothesis 1a: A perceived mastery climate will relate negatively to turnover intention.

In a performance climate, on the other hand, employees’ attention is tuned to social comparison information regardless of whether they initially had an interest in such comparison information (Ames, 1984). Thus, the success criterion in a performance climate is normative ability, and only the best performers are therefore recognized as successful (Ames, 1992b). Such a climate has been found to promote maladaptive outcomes such as turnover intention, controlled motivation, poorer performance, lower persistence, and negative affect (Ntoumanis and Biddle, 1999b; Harwood et al., 2008; Nerstad et al., 2013). While a mastery climate has been shown to be a strong predictor of intrinsic motivation (Cury et al., 1996; Ntoumanis and Biddle, 1999b; Buch et al., 2017), evidence from sports literature has indicated that individuals tend to drop out of their sport when they perceive a high performance climate (Sarrazin et al., 2002). In a work setting, while it is rare for employees to completely drop out of their work, they may become demotivated or consider changing their job (Nerstad et al., 2013). A performance climate is a situation of negative interdependence among colleagues (Ames and Ames, 1984). This means that other colleagues’ rewards or gains will be negatively related to one’s own. Because a performance climate overwhelms employees with information about their colleagues’ performances, such a work situation will stimulate employees’ interest in constantly comparing their own work performance with that of their colleagues (Ames and Ames, 1984; Roberts and Nerstad, 2020). The main focus is on normative abilities and on a constant quest for work success and rewards. Consequently, a performance climate is likely to trigger a motivational shift from doing the job because of the joy of the work itself to doing the job to achieve an external reward or benefit (Vallerand et al., 1986). Employees who experience a performance climate may think about changing their job because they experience reduced intrinsic motivation. We therefore expect the following:

Hypothesis 1b: A perceived performance climate will relate positively to turnover intention.

### Work–Home Spillover

Work–home spillover refers to the transfer of positive and negative experiences between work and home (Kinnunen et al., 2014). It has been defined as the effects of work and home on one another “that generate similarities between the two domains” (Edwards and Rothbard, 2000, p. 180). Previous research has mostly focused on the transfer of similarities in mood, values, skills, and behaviors between the two domains (Rothbard and Dumas, 2012). Spillover further involves two related, yet distinct, sets of concepts (Grzywacz and Marks, 2000). On the one hand, there is negative work–home spillover, which occurs when individuals experience that work has a negative influence on non-work activities due to a lack of the resources needed to manage both work and non-work roles (Grotto and Lyness, 2010). Positive work–home spillover, on the other hand, is characterized by resource enhancement (e.g., Grzywacz et al., 2002). This type of spillover occurs when behaviors and moods acquired in one domain positively affect an individual’s behaviors and moods in another domain (Grzywacz et al., 2007).

A specific focus on both the presence of positive spillover and the absence of negative spillover should facilitate a more accurate assessment of the pathways between variables (Grzywacz and Marks, 2000). The idea of spillover processes in work–home experiences is based on ecological systems theory, in which such experiences are assumed to be a joint function of process, person, and time characteristics (Grzywacz and Marks, 2000). Several meta-analyses indicate that high levels of negative work–home spillover can lead to lower job and life satisfaction, greater general psychological strain, greater somatic or physical symptoms, higher rates of depression, and greater burnout (Kossek and Ozeki, 1998; Allen et al., 2000; Ford et al., 2007). On the other hand, indicators of positive spillover have been associated with greater employee commitment, job satisfaction, family satisfaction, and organizational citizenship behavior (Kossek and Ozeki, 1999; Bragger et al., 2005; McNall et al., 2010). Scholars have also devoted much attention to the influence that negative work–home interfaces have on turnover intention (Boyar et al., 2003; Spector et al., 2007; Carr et al., 2008). Since most Western families are engaged in dual-income arrangements of some sort, they are also concerned with managing both work and non-work activities. When work negatively interferes with non-work, it is likely to be experienced as a role stressor (Spector et al., 2007). Because of younger workers’ relatively high appreciation of leisure as a consequence of increased work pressure and expectations (Twenge et al., 2010), they could perceive negative work–home spillover as a role stressor. Leaving the organization might be a solution to such a role stressor and a means of reducing the experience of negative work–home spillover, thereby allowing the individual to better integrate various life roles (Frone, 2003; Spector et al., 2007; Carr et al., 2008).

### Perceived Motivational Climate and Work–Home Spillover

Several factors have been found to influence work–home spillover. Family friendly benefits, supportive organizational cultures, and job characteristics have all been found to reduce negative spillover (cf. Grotto and Lyness, 2010). Likewise, in a recent meta-analysis of antecedents of positive work–family spillover, Lapierre et al. (2018) found that personal and contextual characteristics of family domain as well as family focused support from supervisors and colleagues, and family friendly work cultures all contributed to increased positive work–home spillover. Contextual characteristics as antecedents to increased positive work–home spillover and reduced negative work–home spillover typically involve those that are resource−providing in nature. Indeed, work climates that promote positive and enriching environments enable facilitation, partly because enriched jobs in combination with supportive work environments promote emotional and intellectual development that can facilitate functioning of another domain (Wayne et al., 2007).

A mastery climate may be representative of an enriching environment for two specific reasons. First, according to the conservation of resources (COR) theory, both environmental and internal processes are involved in stress experiences (Hobfoll, 2001). What people think and do is, in itself, a reflection of contextual processes and scripts (Hobfoll, 2001). Thus, COR theory suggests that resources are largely sociocultural, rather than individualistically framed. Therefore, members who share a work climate can form common perceptions of the available resources (Hobfoll, 2002). Given the mastery climate’s emphasis on aspects such as effort, self-improvement, progress, and cooperation, it represents a pool of resources that are located outside the self and can only be found in individuals’ social domains (Ten Brummelhuis and Bakker, 2012). When provided with these resources, employees who work in a mastery climate may perceive reduced negative work–home spillover because they are better equipped to address the presence of stressors (Ten Brummelhuis and Bakker, 2012). Likewise, the social resources and positive experiences that a perceived mastery climate engenders should increase skills and fulfillment, thus building pathways of positive work–home spillover. As such, a perceived mastery climate may constitute what Greenhaus and Powell (2017) suggested was a life-supportive climate. Such a climate would include single and childless/childfree employees, giving them equal opportunities to focus on their non-work activities. Furthermore, a life-supportive climate would be characterized by employees having a high degree of autonomy to organize their work days, and by opportunities to develop new skills and develop self-confidence that might enhance their participation in other life arenas (Greenhaus and Powell, 2017). This brings us to the second reason why a mastery climate should represent an enriching environment. Through the emphasis on growth processes, including individual control (i.e., what they can do and when they can do it), self-leadership, and self-regulatory strategies, employees who work in a perceived mastery climate are able to decide when they will allocate the resources that enable them to achieve work-related goals and to fulfill their obligations in other areas of life (cf. Ames, 1992a; Pensgaard and Roberts, 2002; Valentini and Rudisill, 2006; Van Ruysseveldt et al., 2011). Indeed, scholars have thoroughly documented the impact that control and autonomy have on the work–home interface. For example, Grotto and Lyness (2010) found that job resources, such as autonomy and skill development, significantly reduced negative work–home spillover. Furthermore, Valcour (2007) found that job complexity and control over work time were positively related to satisfaction with positive work–home interactions. Thus the implicit role of autonomy and control in a perceived mastery climate may represent resources that help employees attain a positive interface between work and non-work roles (cf. Pensgaard and Roberts, 2002; Van Ruysseveldt et al., 2011). In support of this point, researchers have revealed a strong positive relationship between a perceived mastery climate and autonomy (e.g., Ames and Archer, 1988; Ntoumanis and Biddle, 1999a, b; Reinboth and Duda, 2006; Harwood et al., 2008). The available resources in a mastery climate may also be relevant for individuals who are threatened with resource loss, as they should have easier access to coping strategies than do other individuals (Ntoumanis and Biddle, 1999b). A mastery climate offers sources of replaceable resources through its emphasis on learning, autonomy, task variety, reasonable challenges, and belongingness, which are important when threatened with resource loss (cf. Ames, 1992a). Indeed, in organizations in which communication, collaboration, experimentation, and learning are emphasized and acknowledged, employees are more likely to engage in substantial and meaningful work–home-supportive activities because of the employees’ extensive coordination and mutual support (Van Dyne et al., 2007; Greenhaus and Powell, 2017). Therefore, we expect the following:

Hypothesis 2: A perceived mastery climate will relate (a) positively to positive work–home spillover and (b) negatively to negative work–home spillover.

On the other hand, a performance climate entails controlling contextual aspects because of its promotion of interpersonal competition and rivalry, which may lead to negative interdependence among colleagues (Černe et al., 2014; Buch et al., 2015). Employees who are exposed to ambiguous or conflict-producing work environments tend to experience increased tension and negative spillover between work and non-work activities (Greenhaus et al., 1987). Negative emotional spillover from work to non-work activities has previously been found to be the result of stressors at work and career disappointments due to the extensive production of tension and fatigue, which ultimately interfere with employees’ non-work life (Bartolome and Evans, 1980). Furthermore, negative experiences at work can reduce employees’ sense of happiness, well-being, and overall quality of life, and lead to employee alienation (cf. Greenhaus et al., 1987). In other words, working in a performance climate may lead to a resource loss situation that is likely to spill over into the home arena. Indeed, organizations’ focus on measuring productivity in terms of quantity rather than quality, and their emphasis on measurement and control, have been found to create work life difficulties (Rayman et al., 1999). Employees also tend to report work-life difficulties when they experience a sense of inequity in relation to rewards at work, or have a profit-driven focus (Eby et al., 2005). Therefore, we hypothesize the following:

Hypothesis 3: A perceived performance climate will relate (a) negatively to positive work–home spillover and (b) positively to negative work–home spillover.

### The Mediating Role of Work–Home Spillover

We expect that the extent to which a perceived motivational climate influences turnover intention can be partly explained by both negative and positive work–home spillover. We expect that a mastery climate will indirectly reduce turnover intentions by reducing negative spillover due to the inherent resources provided in a mastery climate that should reduce the feeling of stress. We also expect that increased positive spillover will reduce turnover intention because positive spillover can lead to increased positive affect toward both home and work. These assumptions are in line with the dual perspective on work–family spillover (e.g., Carlson et al., 2006; Kinnunen et al., 2006; Wayne et al., 2007), where it is argued that when one role enhances another role, individuals will experience benefits in the receiving role and at the same time attribute these benefits to the sending role. In turn, greater satisfaction is experienced in both domains (cf. Shockley and Singla, 2011) and should ultimately lead to lower intentions to leave an organization. We have argued that working in a mastery climate elicits positive resources that should lead to increased positive and reduced negative work–home spillover. Based on social exchange theory (Blau, 1964) and norms of reciprocity (Gouldner, 1960), individuals who experience positive resources from their environments that also spill over to non-work should eventually have positive behavioral reactions and attitudes toward work in that they try to reciprocate the benefits they have gained from work (Wayne et al., 2007) and feel more obliged to stay with their current employer. We therefore hypothesize the following:

Hypothesis 4: The negative relationship between a perceived mastery climate and turnover intention will be mediated by both (a) negative work–home spillover and (b) positive work–home spillover.

On the other hand, a performance climate should indirectly increase turnover intentions by increasing negative spillover and reducing positive spillover because of the potential resource loss that employees may experience when working in a performance climate. Work pressures, such as an emphasis on profits and competitiveness among colleagues, as well as profit-driven and competitive organizations, have been found to negatively spill over into non-work life because of the strain associated with such work conditions (Wallace, 1997). According to COR theory (Hobfoll, 1989, 2001), stress may occur when employees experience a loss of resources (energy, motivation) at work, which may then spill over into the home domain. In turn, these negative experiences in the home domain may be attributed to the work domain, and an intention to leave the organization may be a way of coping with these stressors. Moreover, in line with spillover theory (Grzywacz and Marks, 2000), negative emotions and behaviors that are built up in the work domain and then transferred to the home domain may determine how employees deal with their work and lead to a wish to seek employment elsewhere in order to attain a sound interface between work and non-work activities. Therefore, we hypothesize:

Hypothesis 5: The positive relationship between a perceived performance climate and turnover intention will be mediated by both (a) negative work–home spillover and (b) positive work–home spillover.

### The Relative Contributions of the Two Spillover Types

Different outcomes of positive and negative spillover have typically been displayed in previous research (Wayne et al., 2015). For example, Aryee et al. (2005) examined the outcomes of negative and positive work–home interfaces and found that negative and positive aspects of the work–home interplay each had different effects on job satisfaction and organizational commitment. Indeed, a vast amount of empirical evidence indicates that increased negative spillover has negative consequences such as lower job and life satisfaction, greater psychological strain, more frequent physical symptoms, higher frequencies of depression, greater burnout, and higher turnover intentions (cf. McNall et al., 2010). Increased positive spillover has typically been related to increased job satisfaction, affective commitment, as well as life satisfaction and positive physical and mental health (cf. Kossek and Ozeki, 1998; Allen et al., 2000; Ford et al., 2007; McNall et al., 2010). The mixed results regarding the relationship between positive work–home interface and turnover intention (cf. McNall et al., 2010) could be explained by the nature of the outcome variable, as turnover intention reflects a negative (rather than positive) outcome. In this study, we investigate these issues further and test the relative contributions of the two spillover types in terms of mediating the relationship between a mastery climate and turnover intention. Although some researchers did not find a significant relationship between positive spillover and turnover intention (e.g., Gordon et al., 2007), other researchers have documented such a relationship (e.g., Moen et al., 2011). We argue that contrasting and accounting for positive spillover as well as negative spillover facilitates a more accurate assessment of the pathways between variables (Grzywacz and Marks, 2000). In turn, it should provide a more accurate view of the role of positive spillover in reducing turnover intention and provide support for the relationship between positive spillover and turnover intention. Nonetheless, we expect that negative spillover should have a stronger influence on turnover than positive spillover. In their review, Baumeister et al. (2001) demonstrated that, when compared to good events, bad events produce larger, more consistent, more multifaceted, and more lasting effects across a broad range of psychological phenomena. Bad events that occur in even small forms, such as everyday situations, have more power than do good events (Baumeister et al., 2001). Such events include the areas of social network patterns, interpersonal interactions, learning processes, and emotions. Furthermore, Baumeister et al. (2001) concluded that the reciprocity of negative affect was especially potent—more influential than that of positive affect. Thus, negative affect and emotional distress may have a stronger influence than do positive affect and pleasant emotions. Similar mechanisms can be found in research on well-being, where job stressors have a stronger influence on negative well-being than on positive well-being (Sonnentag, 2015). Based on the literature review by Baumeister et al. (2001) and on previous studies in which researchers linked various outcomes to positive and negative spillover (e.g., Wayne et al., 2015; Aboobaker and Edward, 2019), it is likely that negative spillover has a more powerful influence on turnover intention than does positive spillover. Thus, we hypothesize the following:

Hypothesis 6: Negative work–home spillover, compared to positive work–home spillover, will have a stronger influence on turnover intention.

## Materials and Methods

### Sample and Procedure

In line with expert advice (Podsakoff et al., 2012), we utilized a two-wave, Web-based questionnaire survey to test the hypotheses and to reduce the potential influence that common-method variance would have on our results. We measured both the predictor variables and the mediator variables at Time 1 and measured the dependent variable at Time 2. We specified a 3-week time lag between the first and second waves. Using a Web-based tool (Confirmit), we sent the survey to 2,800 employees from a Norwegian financial-sector organization. The e-mail included a cover letter with written assurances of confidentiality and aggregate reporting. To assure anonymity (Podsakoff et al., 2003), we informed the respondents that their identifying information—such as e-mail addresses—and responses would be stored separately in encrypted files for data-matching purposes (matching data collected during the two time periods). Furthermore, we informed the respondents that all personal identifying information (e.g., e-mail addresses) would be deleted at a predetermined date. We then asked the respondents to answer the survey questions honestly and assured them that there were no right or wrong answers (Podsakoff et al., 2003). Given that the respondents in our data were nested within leaders, we received an Excel file from the organization with information regarding who was the direct leader of each respondent. Based on this information, we were able to match each respondent with his or her direct leader. The leader’s name was substituted with a leader ID number. The sample size at the leader level was 427.

We received 1,075 completed responses (38%) from the first wave and 838 completed responses (78%) from the second wave. Of the participants, 48% were female. The mean age was 48 years, and the sample consisted of respondents from each age category typically defined in life-span approaches in organizational psychology: ≥39 (settling in adults – Arnett, 2006, *n* = 177), 40−54 (prime working years – Sterns and Huyck, 2001, *n* = 387), 55−62 (approaching retirement – James and Spiro, 2007, *n* = 234), and 63 and above (retirement eligible – James et al., 2011, *n* = 32). Furthermore, 25% held a master’s degree or higher. About 24% reported managerial responsibility, and 98% held a permanent position. Table 1 provides an overview of the other descriptive statistics.

**TABLE 1:** Descriptive Statistics for Key Study Variables (N_*Time* 1_ = 1074; N_*Time* 2_ = 837), Latent Variable Correlations (N_*Time*__1__&__2_ = 1074), and Shared Variance Estimates.

	Mean	SD	1	2	3	4	5	Cronbach’s alpha	Composite reliability
Gender	1.52	0.50							
Age	48.61	9.86							
Education	2.88	0.81							
Leader tenure	3.13	4.01							
Tenure	8.21	9.27							
Managerial responsibility	1.21	0.41							
Work domain	2.97	1.68							
Weekly work hours	40.27	6.88							
Retirement age	65.26	2.53							
(1) Mastery climate	5.18	0.88	**0.57**	0.01	0.07	0.09	0.18	0.85	0.89
(2) Performance climate	3.83	1.26	−0.08**	**0.55**	0.09	0.03	0.01	0.88	0.91
(3) Negative work–home spillover	3.71	1.36	−0.27***	0.30***	**0.67**	0.02	0.13	0.86	0.89
(4) Positive work–home spillover	3.90	1.10	0.30***	0.17***	−0.15***	**0.53**	0.04	0.74	0.77
(5) Turnover intention	2.73	1.45	−0.43***	0.10**	0.36***	−0.20***	**0.77**	0.89	0.94

Scores for mastery climate, performance climate, negative work–home spillover, positive work–home spillover, and turnover intention reflect responses on a 7-point scale ranging from 1 (*strongly disagree*) to 7 (*strongly agree*). Gender: 1 = female, 2 = males; education: 1 = junior high school; 2 = high school; 3 = bachelor’s degree; 4 = master’s degree; 5 = PhD; managerial responsibility: 1 = no, 2 = yes; work domain: 1 = sales, 2 = sales support, 3 = administration, 4 = leadership, 5 = other; latent variable correlations are below the diagonal, squared correlations are above the diagonal, and average variance extracted (AVE) estimates are presented in bold on the diagonal. **p < 0.01 and ***p < 0.001.

### Measures

#### Perceived Motivational Climate

We used 14 items from the motivational climate at work questionnaire (MCWQ) developed by Nerstad et al. (2013). The questionnaire focused on how employees perceived how success was defined in their work situations, and it opened with the following statement: “In my department/work group.” The respondents then assessed the extent to which they perceived that a mastery climate was present (e.g., “Each individual’s learning and development is emphasized,”) and the extent to which they perceived that a performance climate was present (e.g., “Competitive rivalry exists among the employees”). Response options ranged from 1 (*completely disagree*) to 7 (*completely agree*). The Cronbach’s α for a perceived mastery climate was 0.85. For a perceived performance climate, the Cronbach’s α was 0.88.

#### Work–Home Spillover

We used a measure developed by Grzywacz and Marks (2000) to assess work–home spillover. This measure includes four items that assess negative work–home spillover and four items that assess positive work–home spillover. The response options ranged from 1 (*never*) to 5 (*all the time*). Respondents indicated the frequency with which they had experienced each of the items, which included “Your job reduces the effort you can give to activities at home” and “The things you do at work help you to deal with personal and practical issues at home.” The Cronbach’s α values were 0.77 for positive spillover and 0.84 for negative spillover.

#### Turnover Intention

This variable was measured with five items that assess behavioral intent to leave an organization (Kuvaas, 2006). The response options ranged from 1 (*completely disagree*) to 7 (*completely agree*). The items included “I will probably look for a new job in the next year” and “I often think about quitting my present job.” The Cronbach’s α was 0.89.

#### Control Variables

To control for relevant variables that may extraneously influence the hypothesized relationships, we identified and managed several non-focal variables at Time 1. This is essential for “ensuring the generalizability that allows empirical research to benefit individuals, organizations, and society as a whole” (Bernerth and Aguinis, 2016, p. 230). First, because demographic variables can influence differential needs in life (Bagger and Li, 2014), we asked the participants about their gender, age, education, and retirement age, as well as leader and worker tenure, and work domain, because these variables have indicated important influences on the work-home interface in previous research (e.g., Byron, 2005; Valcour, 2007; Moen et al., 2011). Similar results may exist for leader responsibility, which we therefore controlled for as well (measured with the question “*Do you have leader responsibility?*” 1 = *no*; 2 = *yes*). Finally, we controlled for weekly work hours because researchers have frequently shown that this variable is related to perceptions of work–home balance (cf. Valcour, 2007).

### Statistical Analyses

#### Analytical Approach

To determine item retention and secure the variables’ discriminant validity, we performed confirmatory factor analyses (CFA). As our data are ordinal, we applied the weighted least square with a mean-and variance-adjustment (WLSMV) estimator for categorical data using M*plus* 7.3 (Muthén and Muthén, 1998–2014; Brown, 2006). As Jöreskog (2005) emphasized, “ordinal variables are not continuous and should not be treated as if they are” (p. 10). WLSMV is regarded as a robust estimator that provides a precise treatment of categorical data and that does not assume that the variables are normally distributed (Brown, 2006; Rhemtulla et al., 2012). To evaluate the model fit, we applied common guidelines (e.g., the root mean square error of approximation [RMSEA] of <0.08, the comparative fit index [CFI] of >0.95, the Tucker-Lewis index [TLI] of >0.95, and the standardized root mean square residual [SRMR] of <0.10) to evaluate whether there was an acceptable fit (Hu and Bentler, 1999; Marsh et al., 2005). In addition, due to the non-independent observations in the data set, with respondents nested within leaders, we performed a multiple-indicator, multiple-cause (MIMIC) CFA using cluster-robust standard errors at the leader level. This approach is valuable because it enables control over sample heterogeneity (Muthén, 1989).

#### Missing-Value Analysis

Our data included missing values. To explore whether the missing data depended on the variables in the data set, we decided to conduct Little’s (1988) missing-completely at-random (MCAR) test. We applied the SPSS 24 Missing Value Analysis tool with the expectation maximization technique to conduct the analyses. Little’s MCAR test indicated that the data were missing completely at random (i.e., there were no identifiable patterns in the missing data), which can be expressed as follows: χ^2^ (72, *n* = 838) = 78.72, *p* = 0.275. The range of missing data was most severe for turnover intention, which was measured at Time 2 (22% missing values). Among the other study variables, we only found a few missing values among the demographic variables (e.g., employment: 1.2% missing values). Little’s MCAR test indicated that the data were missing completely at random, as the *p*-Value was non-significant.

#### Handling Missing Data

We handled the missing values that occurred in this study based on Newman’s (2014) five practical guidelines for handling missing data^1^. First, we removed one case from the data set because information about the direct leader was missing. The resultant data set comprised N_*Time*__1_ = 1074 and N_*time*__2_ = 837. Given that missing values in item responses occurred mainly on Time 2 for the turnover intention scale (up to 22%), we applied a multiple imputation procedure using M*plus* (i.e., Newman, 2014). This procedure assumes that missing values occurred randomly (Enders, 2010). We followed the recommendations by Enders (2017) and conducted multiple imputation with chained equations. We conducted the H0 imputation procedure in M*plus* (Asparouhov and Muthén, 2010; Enders, 2017), and we generated *m* = 20 complete data sets (Graham et al., 2007). All further analyses were based on these 20 data sets. In addition, we pooled the model parameters by applying Rubin’s combination rules (Enders, 2010). The pooling also included the model fit statistics (Enders and Mansolf, 2016).

## Results

### Descriptive Statistics

Table 1 presents the descriptive statistics and latent variable correlations for the variables under investigation. We decided to follow the recommendations by Farrell (2010) suggesting that the confirmatory factor analysis (CFA) correlation matrix is a preferred option when it comes to assessing discriminant validity because it accounts for measurement error. Further, we tested reliability using three indicators: Cronbach’s alpha (Cronbach, 1951), composite reliability (CR; Fornell and Larcker, 1981), and average variance extracted (AVE; Fornell and Larcker, 1981). The results of the reliability analysis indicate that all included concepts had overall adequate reliability, as the Cronbach’s α of the various measures ranged from 0.74 to 0.89, the CRs ranged from 0.77 to 0.94, and the AVEs ranged from 0.53 to 0.77 (see Table 1). We also compared the AVEs of the latent variables to the squared correlations with the other latent variables to assess the discriminant validity of the constructs. As Table 1 indicates, we found support for discriminant validity because the AVEs of the latent variables were all larger than the squared correlations with the other variables.

### Confirmatory Factor Analysis Results

Our research model consisted of five latent variables (i.e., a correlated-traits model): a mastery climate, a performance climate, negative work–home spillover, positive work–home spillover, and turnover intention. However, in the CFA, we also controlled for various control variables (gender, age, education, retirement age, leader tenure, employee tenure, work domain, employment, managerial responsibility, and weekly work hours). Since modification indices are not available for multiple imputation, we first conducted a CFA *without* multiple imputation, χ^2^ (175) = 1118.58, *p* < 0.001, χ^2^/*df* = 3.24, RMSEA = 0.053, CFI = 0.964, and TLI = 0.956. The modification indices of the CFA suggested the existence of severe cross-loadings for item 3 (“Having a good day at work makes you a better companion when you get home”) between positive work–home spillover and negative work–home spillover. Further, the factor loading of item 3 was 0.36, which is lower than the commonly recommended criterion of 0.50 (Nunnally and Bernstein, 2007). We therefore removed item 3 from further analyses that utilized multiple imputation. In the next step, we conducted a CFA based on multiple imputation in which the results indicated an acceptable model fit, χ^2^ (255) = 1104.25; χ^2^/*df* = 4.33, RMSEA = 0.056, CFI = 0.966, TLI = 0.958. To be certain that the five-factor model did fit the data better than other alternative models, we tested alternative models, such as one in which all variables loaded on a common factor [χ^2^ (288) = 8116.09; χ^2^/*df* = 28.18, RMSEA = 0.159, CFI = 0.685, TLI = 0.655], as well as a three-factor model in which negative and positive work–home spillover loaded on the same factor [χ^2^ (267) = 2947.67; χ^2^/*df* = 11.03, RMSEA = 0.097, CFI = 0.892, TLI = 0.873]. Since the fit of such alternative models was less acceptable than the five-factor model, the five-factor model (mastery climate, performance climate, turnover intention, negative work–home spillover, and positive work life spillover) formed the basis for further analyses. In addition, all factor loadings were sufficiently high (ranging from 0.66 to 0.94) and exceeded the common criterion of 0.50 (Nunnally and Bernstein, 2007).

### Structural Equation Modeling Results

Given the nested nature of our data, we tested our predicted mediation model using structural equation modeling (SEM) with cluster robust standard errors at the leader level. For Hypothesis 1, we predicted there would be (a) a direct negative relationship between a perceived mastery climate and turnover intention, and (b) a positive relationship between a performance climate and turnover intention. The results were in line with these predictions (a) β = −0.423, *SE* = 0.031, *p* < 0.001, and 95% CI (−0.484, −0.362) and (b) β = 0.107, *SE* = 0.037, *p* < 0.01, and 95% CI (0.036, 0.179), thus supporting Hypothesis 1a and 1b.

We then tested the predicted mediation model, including the direct relationship between a perceived mastery climate and work–home spillover. The results indicated an acceptable overall model fit, χ^2^ (480) = 2146.19, χ^2^/*df* = 4.47, RMSEA = 0.057, CFI = 0.943, and TLI = 0.934^2^. As shown in Table 2, we found support for Hypothesis 2, thus predicting a positive relationship between a perceived mastery climate and (a) positive work–home spillover (β = 0.328, *SE* = 0.033, *p* < 0.001), as well as a negative relationship with (b) negative work–home spillover (β = −0.287, *SE* = 0.030, *p* < 0.001). We only found partial support for Hypothesis 3 (see Table 2), which predicted a negative relationship between a performance climate and (a) positive work-life spillover (β = 0.146, *SE* = 0.030, *p* < 0.001), and a positive relationship between a perceived performance climate and (b) negative work–home spillover (β = 0.281, *SE* = 0.026, *p* < 0.001). To our surprise, the relationship between a performance climate and positive work-life spillover was *positive* rather than negative, thus only partly supporting Hypothesis 3.

**TABLE 2:** Structural Equation Modeling Results for the Direct Relationship between the Perceived Motivational Climate and Turnover Intention and the Mediation Model.

Variables	Parameter estimate
**Dependent variable: turnover intention**	
Mastery climate	−0.423(0.031)***
Performance climate	0.107(0.037)**
Gender	−0.034(0.032)
Age	−0.264(0.033)***
Education	0.124(0.035)***
Leader tenure	−0.051(0.035)
Tenure	0.061 (0.035)
Managerial responsibility	0.023 (0.035)
Work domain	0.044 (0.039)
Weekly work hours	−0.021(0.033)
**Dependent variable: negative work–home spillover**	
Mastery climate (*a*_1_)	−0.287(0.030)***
Performance climate (*a*_3_)	0.310(0.029)***
Gender	−0.107(0.031)***
Age	−0.112(0.035)***
Education	0.040 (0.035)
Leader tenure	−0.065(0.030)*
Tenure	0.077(0.030)**
Managerial responsibility	0.075(0.036)*
Work domain	−0.006(0.031)
Weekly work hours	0.139(0.033)***
Retirement age	−0.044(0.030)
**Dependent variable: positive work–home spillover**	
Mastery climate (*a*_2_)	0.328(0.033)***
Performance climate (*a*_4_)	0.162(0.033)***
Gender	−0.173(0.033)***
Age	−0.017(0.038)
Education	0.080(0.039)*
Leader tenure	−0.032(0.036)
Tenure	0.006 (0.038)
Managerial responsibility	0.058 (0.040)
Work domain	0.055 (0.038)
Weekly work hours	0.031 (0.040)
Retirement age	−0.013(0.035)
**Dependent variable: turnover intention**	
Negative work-home spillover (*b*_1_)	0.244(0.039)***
Positive work-home spillover (*b*_2_)	−0.089(0.036)*
Mastery climate	−0.325(0.038)***
Performance climate	0.046 (0.037)
Gender	−0.023(0.032)
Age	−0.237(0.032)***
Education	0.121(0.034)***
Leader tenure	−0.037(0.034)
Tenure	0.042 (0.034)
Managerial responsibility	0.010 (0.033)
Work domain	0.051 (0.039)
Retirement age	−0.051(0.033)
**Mediation test^1^**	
*a*_1_ * *b*_1_	−0.094(0.020)***
*a*_2_ * *b*_2_	−0.039(0.017)*
*a*_3_* *b*_1_	0.103(0.020)***
*a*_4_ * *b*_2_	−0.019(0.009)*
***Contrast: Negative work-home spillover vs. Positive work-home spillover***	−0.055(0.022)**

Gender: 1 = female, 2 = male; education: 1 = junior high school; 2 = high school; 3 = bachelor’s degree; 4 = master’s degree; 5 = PhD; managerial responsibility: 1 = no, 2 = yes; work domain: 1 = sales, 2 = sales support, 3 = administration, 4 = leadership, 5 = other. ^1^For the mediation test, the unstandardized values are presented, while all other values in the table are standardized. *p < 0.05; **p < 0.01; and ***p < 0.001.

**FIGURE 1:**
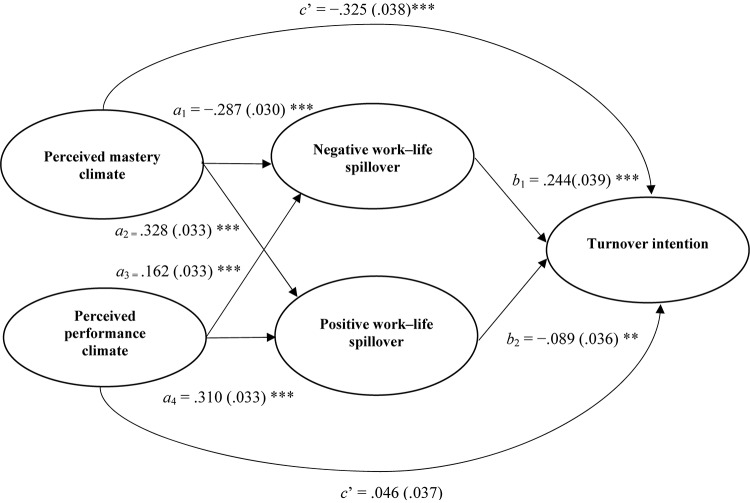
Results of the predicted mediation model. The figure gives a simplified presentation of the tested model. The numbers in parentheses are standard errors. *a*, influence of independent variable on mediator; *b*, influence of mediator on dependent variable; *c*’, direct influence. **p < 0.01 and ***p < 0.001.

As predicted in Hypotheses 4a and 4b, both positive and negative work–home spillover partly mediated the negative relationship between a perceived mastery climate and turnover intention (see Figure 1 and Table 2). This was indicated by the significantly reduced direct influence that a perceived mastery climate had on turnover intention (β = −0.325, *SE* = 0.038, *p* < 0.001, and 95% CI [−0.399, −0.251]). The significant indirect influence of a mastery climate on turnover intention through negative work–home spillover (β = −0.094, *SE* = 0.020, *p* < 0.001, 95% CI [−0.132, −0.056]) and positive work–home spillover (β = −0.039, *SE* = 0.017, *p* < 0.01, 95% CI [−0.073, −0.005]), combined with the fact that the confidence intervals did not include zero, indicates additional support for Hypotheses 4a and 4b (see Table 2).

Further, the results indicated a significant, positive direct relationship between negative work–home spillover and turnover intention, β = 0.244, *SE* = 0.039, *p* < 0.001, and 95% CI (0.168, 0.321), as well as a significant, negative direct relationship between positive work–home spillover and turnover intention, β = −0.089, *SE* = 0.036, *p* < 0.001, and 95% CI (−0.160, −0.017).

Hypotheses 5a and 5b predicted that both positive and negative work–home spillover would mediate the positive relationship between a perceived performance climate and turnover intention. Although the significant direct influence that a perceived performance climate had on turnover intention became insignificant when the predicted mediators were included in the model, we did not find support for Hypothesis 5 given that the confidence interval included zero (β = 0.046, SE = 0.037, *p* > 0.05, and 95% CI [−0.027, 0.120]) (MacKinnon et al., 2002). However, as shown in Figure 1 and Table 2, our results indicated that a performance climate had a significant indirect influence on turnover intention through negative work–home spillover (β = 0.103, *SE* = 0.020, *p* < 0.001, 95% CI [0.063, 0.142]) and positive work–home spillover (β = −0.019, *SE* = 0.009, *p* < 0.05, 95% CI [−0.037, −0.002]) (MacKinnon et al., 2002).

We also conducted contrast analyses (see Table 2) to identify each mediator’s unique abilities to account for the influence that a perceived mastery climate would have on turnover intention (Preacher and Hayes, 2004). The confidence interval of the contrast analysis did not contain zero, 95% CI [−0.098, −0.012], which suggests that the contrast was significant. Therefore, we were able to compare the strength of the individual indirect influences (negative and positive spillover). In support of Hypothesis 6, negative spillover had a significantly greater indirect influence than positive spillover on turnover intention.

## Discussion

In this study, we explored whether the relationship between a perceived motivational climate and turnover intention can be explained in part by work–home spillover. In line with our main hypotheses, a perceived mastery climate was negatively related to turnover intention, positively related to positive work–home spillover, and negatively related to negative work home spillover. A perceived performance climate was positively related to turnover intention and positively related to negative work–home spillover. Contrary to our expectations, a performance climate was also positively related to positive work–home spillover. Furthermore, negative and positive work–home spillover each mediated the relationship between a perceived motivational climate and turnover intention. Finally, our results showed that negative spillover had a stronger direct influence on turnover intention than positive spillover. Accordingly, negative spillover should be given greater credence than positive spillover when trying to reduce turnover intention. As such, a perceived mastery climate is of particular importance because it plays a critical role in reducing negative spillover.

### Theoretical Implications

Our findings indicate that a perceived motivational climate influences turnover intention through positive and negative work–home spillover. Previous research (Nerstad et al., 2013) has supported the view that a perceived mastery climate reduces turnover intention, and that a performance climate increases turnover intention. We offer further elaboration on this issue by providing insight into the mechanisms by which a motivational climate operates.

Employees working in an organization with a mastery climate are likely to continue working in that organization, partly due to that climate’s positive consequences. Indeed, the social resources and positive experiences that a perceived mastery climate engenders reduce tensions between work–home and thus build pathways of positive work–home spillover. Thus, the characteristics of a mastery climate seem to serve a positive function by creating positive work–home spillover. Therefore, we contribute to the advancement of the research on work-climate influences by moving beyond specific family friendly climates when predicting work–home spillover. Our results are similar to those of other researchers who investigated the impact that climate variables have on the work–home interface (cf. Aryee et al., 2013; Bagger and Li, 2014). A mastery climate represents a pool of resources that are available to members; these resources significantly influence perceptions of work–home spillover. Our finding that a perceived mastery climate reduces negative spillover also aligns well with previous research, showing that the resources apparent in a perceived mastery climate increase the likelihood of successful goal achievement at lower psychological costs, thus reducing work stress (Van Ruysseveldt et al., 2011). This may indicate that the resources present in a mastery climate help members cope with difficulties concerning work–home issues at work. The resources offered in a mastery climate can also create positive experiences that members bring with them to other life domains via emotional contagion (e.g., Bakker et al., 2005, 2008), which correlates with our finding that a perceived mastery climate increases positive work–home spillover.

Thus, our findings extend previous research on the work–home interface by exploring the influence of a motivational climate on work–home spillover. As a motivational climate can exist, regardless of specific family friendly climates, we were able to examine other dimensions of the organizational climate when predicting the quality of the interface between work and non-work. Work-climate dimensions, such as role stress, autonomy, work-group cooperation, friendliness, and warmth, are relevant for the perception of positive work–home spillover (Cleveland et al., 2005). Still, to date, most research on life-friendly climates has focused on specific family supportive characteristics. Our findings may encourage both researchers and organizations to focus on the existing norms that define commitment, success, and appropriate behaviors that foster work–home spillover, in line with Friedman and Johnson (1996).

An interesting finding in the current study that is suitable for future exploration is the finding related to performance climate. Our results indicate that a performance climate increases negative spillover between work and family. A performance climate facilitates work pressures, such as an emphasis on profits, competitiveness among colleagues, and work overload, all of which contribute to negative spillover between work and family (Wallace, 1997). However, we also found that performance climate slightly increases the positive spillover between work and family. One explanation for this may be that the positive repercussions of a mastery climate balance out the negative consequences of a performance climate, thereby reducing the negative impact of the performance climate. This aligns well with our finding that performance climate and mastery climate are orthogonal (uncorrelated), and with previous research showing that a mastery climate reduces the negative influence of a performance climate (Ommundsen and Roberts, 1999; Černe et al., 2014; Buch et al., 2015). However, previous research has also indicated that mastery and performance climates are negatively related (e.g., Nerstad et al., 2013; Černe et al., 2014). Thus, future studies should explore the nature of the relationship between mastery and performance climates. For example, it would be interesting to elaborate on the findings of Ommundsen and Roberts (1999) as regards different climate profiles. In work climates where the improvement of results and profits is essential to stay in business, we need to increase our knowledge of how organizations and managers can cope with such demands, while at the same time focusing on developmental processes and shared perceptions of growth (i.e., mastery climate) among employees. For example, in our study, the mean levels of performance climate are lower than the mean levels of mastery climate. In light of Ommundsen and Roberts (1999) findings regarding different climate profiles, it may be that lower levels of performance climate combined with higher levels of mastery climate motivate some employees (e.g., extrinsically motivated employees) (Harwood et al., 2015). The participants in our study all belong to a financial sector where the improvement of results and profits is essential to stay in business. Thus, the influence of different climate profiles in different sectors could also be an interesting avenue for future research^3^.

Our results further indicated that, compared to positive work–home spillover, negative work–home spillover seems to have a stronger direct influence on turnover intention. This is in line with previous research, which has typically displayed a stronger link between positive spillover and positive work attitudes than between positive spillover and negative work attitudes (Aryee et al., 2005; Wayne et al., 2015). These results may be indicative of the differing impacts that bad and good effects have on psychological phenomena (Baumeister et al., 2001). As such, our findings contribute to explaining the mixed results regarding the link between positive work–home spillover and turnover intention (cf. McNall et al., 2010). In fact, this may be a result of the relative power of bad versus good in predicting reduced negative outcomes.

### Practical Implications

Reducing turnover intention is important for many reasons. Obviously, the act of replacing employees is costly; in addition, high turnover can influence the stability, quality, and consistency of organizations’ products or services (Trevor and Nyberg, 2008). Given both the increased number of families who rely on dual incomes and the changing appreciation of leisure among younger employees (Twenge et al., 2010), organizations’ primary focus should be to find ways to retain these employees. Today’s workforce relies on employers that facilitate the interface between work life and other life arenas. When negative interfaces arise between work and non-work, negative consequences may result for both organizations and individuals, including effects on their physical and psychological health (Eby et al., 2005). Thus, employees may become motivated to seek employment elsewhere if their current employers do not help remedy work–home interference. A performance climate seems to have negative consequences for individuals in that they experience negative work–home spillover, leading to them having an increased intention to leave their employer. Although we also found a significant positive relationship between a performance climate and positive work–home spillover, its positive relationship with negative work–home spillover should be a warning to organizations to reduce the growth of a performance climate. Employees working in a perceived mastery climate experience increased positive work–home spillover and reduced negative work–home spillover and are thus less likely to want to quit. Organizations could therefore benefit from creating mastery climates for their employees. This assertion is particularly evident because a perceived mastery climate plays a crucial role in reducing negative spillover, which has the strongest influence on turnover intention. Previous research (cf. Ames, 1992a, b; Nerstad et al., 2017) has indicated that important practices in creating a mastery climate include ensuring employees’ autonomy and recognizing employees’ progress improvement and self-referenced ability. Furthermore, actions that emphasize the value of helping behavior are important, as are actions that ensure employees have the time and opportunity for growth (Ames, 1992a, b; Nerstad et al., 2017). Furthermore, previous research has also indicated that leader behavior (Pensgaard and Roberts, 2002) and a commitment-based human resource management climate (Nerstad, 2012) are vital to the facilitation of a mastery climate.

### Limitations and Future Directions

Despite the significance of our findings, these results must be interpreted in terms of the research’s limitations. First, because our results indicate that work–home spillover only partially mediates the relationship between a perceived mastery climate and turnover intention, other factors may be in play. For example, as we were primarily concerned with the work–home interface, regardless of marital and parental status, we did not control for those variables. However, it would be interesting for future studies to include these variables and thus investigate whether some of the relationships addressed in this study are more relevant to certain groups of employees than to others. In addition, facet-specific climates may coexist, and, as such, they may influence one another and change the resulting consequences. Future studies should explore how parallel climates (e.g., a mastery climate and a family supportive climate) interact to influence work–home spillover. Another interesting avenue of research in this regard would be to investigate macroclimates—particularly how they influence facet-specific climates in facilitating a positive work–home interface. Furthermore, several studies have indicated that home-supportive practices, such as flexible working arrangements, buffer the consequences of negative spillover between work and home (e.g., Batt and Valcour, 2003; Wang and Walumbwa, 2007). Given that the success of home-supportive practices likely depends on contextual factors, a climate’s success in increasing positive work–home spillover is also likely dependent on home-supportive practices. Therefore, further research is required to determine how the interplay between home-supportive practices and contextual factors influences the work–home interface. Related to this is the finding from previous research that positive and negative spillover have distinct antecedents and different effects on outcomes (Aryee et al., 2005; Wayne et al., 2015), such as turnover intentions. Future studies could therefore address this by including both several microclimates and home-supportive practices to investigate their relative contributions to positive and negative spillover. Second, there is also the issue of determining why a perceived mastery climate increases positive work–home spillover and reduces negative work–home spillover. In this study, we discussed what we assumed to be the theoretical links that explain these relationships. For example, we argued that a perceived mastery climate that promotes growth, learning, and autonomy can influence work–home spillover. However, we did not empirically test these assumptions. Therefore, future studies should include relevant variables, such as control over one’s work schedule (i.e., work autonomy), to empirically investigate the theoretical assumptions we made in this study. Third, future research could also focus on comparative research among generations. Our sample consisted of respondents representing each age category typically defined in life-span approaches: 21−39 (settling in adults – Arnett, 2006), 40−54 (prime working years – Sterns and Huyck, 2001), 55−62 (approaching retirement – James and Spiro, 2007), and 63 and above (retirement eligible – James et al., 2011). However, the number of respondents in each group could be increased in order to clarify whether the importance of increased positive and reduced negative spillover between work and home to turnover intention differs across generations. Fourth, although our cross-lagged design provided an improvement compared to a cross-sectional design, we were not in a position to draw conclusions with respect to the causal order of our variables. In addition, our data would have been more potent if we had employed a longer span between the various data collections or even employed a longitudinal design in which all of the variables were investigated at multiple points. To remedy these shortcomings, future studies should engage other methods and methodological procedures.

One interesting avenue of methodological procedures is the episodic approach that Maertz and Boyar (2011) demonstrated. They argued that the levels approach is less suitable for studying the work–home interface because interrole incompatibilities begin at a specific time and place and thus need to be considered from a short-term perspective. In addition, spillover processes can be experienced without a specific causal direction, as the levels approach suggests (Maertz and Boyar, 2011). Maertz and Boyar (2011) argued for the use of an episodic approach because it enables precise attributions of cause and more accurate memory recall. Finally, we treated the experience of work–home spillover as an individual psychological concept. Individuals’ own perceptions of spillover are naturally important, but they also isolate individuals from the organizations and the homes to which they belong (Grzywacz and Carlson, 2007). This raises the issue of whether work–home spillover is a psychological or social construct and whether one can claim positive spillover due to an individual experience, even if individuals in another domain (e.g., family members, friends) disagree (Grzywacz and Carlson, 2007). Thus, future researchers could benefit from including the opinions of other parties (such as employers and families and friends) when examining the work–home interface. Related to this is a future focus on life-to-work influences. Our primary concern in this paper was to show how contextual variables specific to work environments spill over to other life domains, which is why we chose to focus on the work-to-life direction. However, bidirectional influences are indeed important and should be included in future research.

## Conclusion

Despite these limitations, the present research makes some important contributions. First, we responded to the calls to enrich our knowledge about contextual influences on work–home spillover and its outcomes (cf., Burke, 2012; Major and Germano, 2012) by providing support for the view that a perceived motivational climate influences perceptions of positive and negative work–home spillover. Second, our results indicate that turnover intention could result from the influences of a motivational climate and work–home spillover, and that negative spillover has the strongest direct influence on turnover intention. Thus, by contrasting the influence of positive versus negative spillover on turnover intention, we contribute to further clarification of the link between positive work–home spillover and turnover intention (cf., McNall et al., 2010). Our results emphasize the influence that contextual factors have on work–home spillover, indicating that work–home spillover is not solely in the eye of the beholder but is, in fact, a result of the social domain. This research provides valuable insight for organizations that wish to promote healthy and life-supportive environments.

## Data Availability Statement

The datasets for this study are not available due to the regulations set by the Norwegian Center for Research Data. This is to protect respondent confidentiality and participant privacy.

## Ethics Statement

The studies involving human participants were reviewed and approved by the NSD − Norwegian Centre for Research Data. The patients/participants provided their written informed consent to participate in this study.

## Author Contributions

KK and CN contributed to the conception and design of the study. CN organized the database, performed the statistical analysis, and wrote sections of the manuscript. KK wrote the first draft of the manuscript. AD wrote sections of the discussion. KK, CN, and AD contributed to the manuscript revision, read, and approved the submitted version.

## Conflict of Interest

The authors declare that the research was conducted in the absence of any commercial or financial relationships that could be construed as a potential conflict of interest.

## Footnotes

1 We would like to thank one of the reviewers for this suggestion.

2 It should be noted that the model fit was better when a performance climate was excluded from the model: χ^2^ (256) = 1056.76, χ^2^/*df* = 3.24, RMSEA = 0.054, CFI = 0.968, and TLI = 0.960. However, as requested by one of the reviewers, we retained a performance climate in the final model.

3 We would like to thank one of the reviewers for fruitful discussions concerning this matter.
